# CGH analysis of ductal carcinoma of the breast with basaloid/myoepithelial cell differentiation

**DOI:** 10.1054/bjoc.2001.1869

**Published:** 2001-08

**Authors:** C Jones, A V Nonni, L Fulford, S Merrett, R Chaggar, V Eusebi, S R Lakhani

**Affiliations:** The Ludwig Institute for Cancer Research/UCL Breast Molecular Pathology Group, Royal Free & University College Medical School, UK; Department of Pathology, University of loannina Medical School, Greece; Department of Histopathology, Royal Free & University College Medical School, London, UK; Department of Oncology, University of Bologna, Italy

**Keywords:** CGH, myoepithelial, ductal carcinoma, breast, keratin 14, basal cells

## Abstract

2–18% of ductal carcinoma-No Special Type (NST) are reported to express basal cell keratin 14 and such tumours may have a different metastatic pattern and prognosis. We performed immunohistochemistry for cytokeratins 19 (luminal) and 14 (basal) on 92 ductal carcinoma-NST. Those tumours showing CK14 expression were further characterized by immunohistochemistry for myoepithelial cell phenotype and analysed by comparative genomic hybridization. The 7 cases of ductal carcinoma-NST exhibiting a basal cell phenotype were all grade III tumours and showed a molecular cytogenetic profile similar to more conventional myoepithelial cell carcinomas. Therefore it appears that grade III invasive ductal carcinomas contain a subset of tumours with specific morphological and cytogenetic characteristics, and probably prognosis for the patient. © 2001 Cancer Research Campaign http://www.bjcancer.com


*British Journal of Cancer* (2001) **85**(3), 422–427
© 2001 Cancer Research Campaign
doi: 10.1054/ bjoc.2001.1869, available online at http://www.idealibrary.com on IDEAL®

http://www.bjcancer.com

### CGH analysis of ductal carcinoma of the breast with basaloid/myoepithelial cell differentiation

**C Jones[1*], AV Nonni[2], L Fulford[3], S Merrett[1], R Chaggar[1], V Eusebi[4] and SR Lakhani[1,3*]**

[1]The Ludwig Institute for Cancer Research/UCL Breast Molecular Pathology Group, Royal Free & University College Medical School, UK; [2]Department of Pathology, University of Ioannina Medical School, Greece; [3]Department of Histopathology, Royal Free & University College Medical School, London, UK; [4]Department of Oncology, University of Bologna, Italy

**Summary** 2–18% of ductal carcinoma-No Special Type (NST) are reported to express basal cell keratin 14 and such tumours may have a different metastatic pattern and prognosis. We performed immunohistochemistry for cytokeratins 19 (luminal) and 14 (basal) on 92 ductal carcinoma-NST. Those tumours showing CK14 expression were further characterized by immunohistochemistry for myoepithelial cell phenotype and analysed by comparative genomic hybridization. The 7 cases of ductal carcinoma-NST exhibiting a basal cell phenotype were all grade III tumours and showed a molecular cytogenetic profile similar to more conventional myoepithelial cell carcinomas. Therefore it appears that grade III invasive ductal carcinomas contain a subset of tumours with specific morphological and cytogenetic characteristics, and probably prognosis for the patient. © 2001 Cancer Research Campaign http://www.bjcancer.com

**Keywords**: CGH; myoepithelial; ductal carcinoma; breast; keratin 14; basal cells

The ducts and lobules of the normal human mammary gland are lined by 2 epithelial cell types, luminal and basal (myoepithelial) cells. Previous work (Lakhani et al, 1999) has demonstrated that LOH identified in invasive carcinoma is already present independently in 'normal' luminal and myoepithelial cells. This argues for a common precursor cell which must have acquired the genetic alteration prior to differentiation into the 2 epithelial cell types. It is intriguing therefore that myoepithelial cells transform so rarely, and that myoepithelial cell carcinomas of the breast appear to be so rare in clinical practice (Tavassoli, 1991; Damiani et al, 1997; Foschini and Eusebi, 1998). References in the literature are generally composed of single case reports (Erlandson and Rosen, 1982; Thorner et al, 1986; Eusebi et al, 1987; Accurso et al, 1990; Desautels, 1990; Lakhani et al, 1995; Shiraishi et al, 1999). A comparative genomic hybridization (CGH) study of 10 cases of conventional myoepithelial cell carcinoma demonstrated that these aggressive tumours are genetically different from invasive ductal carcinoma-NST in having very few, but specific genetic alterations (Jones et al, 2000).

The contribution of myoepithelial cells to 'ordinary' ductal carcinomas is unclear, and reports suggest that 2–18% of so-called invasive ductal carcinomas-NST show focal or diffuse myoepithelial cell differentiation immunohistochemically, using a range of markers including basal cell cytokeratins, actin, calponin, caldesmone, S100 protein (Gusterson et al, 1982; Nagle et al, 1986; Dairkee et al, 1988; Guelstein et al, 1988; Gould et al, 1990; Wetzels et al, 1991; Malzahn et al, 1998; Tsuda et al, 1999). A recent study utilizing cDNA microarrays used a hierarchical clustering method to group breast tumours according to their similarity in patterns of gene expression, and found a 'basal-like' group consisting of 15% of tumours analysed (Perou et al, 2000). Tumours with a basal cell phenotype have been reported to have a different metastatic pattern and prognosis (Tsuda et al, 2000)

In the present study we aimed to study the molecular cytogenetic profile of invasive carcinomas exhibiting a basal/myoepithelial phenotype by laser capture microdissection (LCM) and CGH, and compare the data to that of pure myoepithelial cell carcinomas and 'ordinary' invasive ductal carcinomas-NST in order to investigate whether their immunohistochemical phenotype is reflected at a molecular genetic level.

### MATERIALS AND METHODS

91 cases of invasive ductal carcinomas-NST were chosen at random from the files of the Histopathology Department, University College Hospital, London, UK. These were 6 grade I, 45 grade II, and 41 grade III according to the grading system used in the UK Breast Screening Programme (NCGBSP, 1995). Sections from formalin-fixed, paraffin-embedded tissues were stained immunohistochemically for the luminal epithelial marker cytokeratin (CK) 19 and the basal cell keratin CK14. Those tumours (6 cases – 6.6%) showing a focal (one or more small areas of keratin 14-positive cells) or diffuse CK14 expression, together with a consultation case sent to SRL, which was diffusely positive with keratin 14, were selected and here defined as cases with basal cell phenotype.

These were further characterized immunohistochemically by staining with calponin, caldesmone, smooth muscle actin (SMA), and S100 protein which are markers of myoepithelial cells (Foschini et al, 2000). Immunohistochemistry for oestrogen receptor (ER), progesterone receptor (PR), p53 and c-erbB2 was also carried out on all CK14-positive tumours and the age-matched controls. Hence a

*Received 10 January 2001*
*Revised 4 April 2001*
*Accepted 5 April 2001*

*Correspondence to:* SR Lakhani

* Current address: The Breakthrough Toby Robins Breast Cancer Research Centre, Institute of Cancer Research, London UK.

total of 7 cases (of a total of 92) with a basal cell phenotype were studied (source and dilutions for all of the antibodies used are reported in Table 1).

All sections were dewaxed in xylene, taken to absolute alcohol (74OP) and blocked for endogenous peroxidase with 3% hydrogen peroxidase in methanol for 10 mins. Sections were rinsed in tap water and subjected to the appropriate pre-treatments as in Table 1. The sections were blocked in normal goat serum (1/10 in Tris buffered saline) for 10 minutes and the primary antibodies were applied for 1 hour. The primary antibodies were rinsed off in 0.05% Tween 20 in Tris buffered saline (TBS/Tween), developed using Dako Duet/HRP system and visualized with DAB (Kem-em-Tec).

The scoring system used for assessment of ER and PR was the Quick Score method (Barnes et al, 1996). c-erbB2 was scored according to the guidelines published by Ellis and co-workers (Ellis et al, 2000). The p53 was scored positive if greater than 10% of the cells showed strong nuclear staining.

Cases exhibiting a basal cell phenotype, as well as age- and grade-matched invasive ductal carcinoma NST showing no CK14 immunoreactivity, were analysed by comparative genomic hybridization (CGH). Some of the tumours (4 cases) showing a basal cell phenotype were heterogeneous in their staining in the sense that areas containing numerous keratin 14-positive cells were alternating with patches of negative cells and hence, in these cases, CK14-positive and -negative areas were microdissected from the same case. Formalin-fixed, paraffin-embedded sections were microdissected using Laser Capture Microdissection (LCM) using the PixCell II system (Arcturus, Mountain View, California). DNA extraction and CGH analysis was carried out as described previously (Jones et al, 2000). Briefly, the DNA was extracted with 0.5 μg $\mu l^{-1}$ proteinase K. Amplification and fluorescent labelling of the DNA from microdissected tumour and normal tissue was carried out by DOP-PCR in 2 rounds as previously published (Wells et al, 1999) and hybridized to normal male metaphase spreads (Vysis UK Ltd, Richmond, England) for 2–3 days at 37°C. Metaphase chromosome preparations were captured using a Zeiss Axioskop microscope, Photometrics (Munich, Germany) KAF1400 CCD camera, and Vysis SmartCapture software. Image analysis was performed using Vysis Quips CGH software. Between 5 and 10 representative images of high quality hybridizations were analysed, and the results combined to produce an average fluorescence ratio for each chromosome. Control experiments were carried out using normal:normal (microdissected normal lymph node) co-hybridizations, whose average red:green ratio levels and 95% confidence intervals were used to set the lower and upper limits for scoring losses and gains of genetic material as 0.80–1.20. The comparison of the mean number of changes between CK14 positive and CK14 negative was done using a one-sided Student's *t*-test.

### RESULTS

The 7 tumours that stained positive for keratin CK14 by immunohistochemistry were from patients with an age range of 44 to 87 years. The tumours were large ranging from 1.5 to 10 cm (mean 3.7 cm). They displayed a combination of pushing and invasive margins and were arranged in large sheets, solid nests or trabeculae of round to polygonal cells showing round to ovoid irregular nuclei with large nucleoli (Figure 1). Their cytoplasm was eosinophilic without evidence of granularity. No glandular structures were evident and necrosis was a common feature (5 of 7 cases). Mitoses, including abnormal forms, exceeded 8 per 10 high power fields. There was a moderate to prominent lymphoplasmacytic infiltrate at the borders of 5 out of 7 tumour. Therefore all these lesions were included in the spectrum of grade III invasive ductal carcinoma.

Immunohistochemistry (Table 2) with keratin 14 antibody stained more than 50% of cells in 3 of 7 tumours (cases 2, 3 and 6). In the remaining 4 cases the same antibody decorated 10–20% of cells. 2 cases were negative with keratin 19 while the other 5 were positive. The keratin 14-positive tumours were also positive with at least 2 other myoepithelial markers 4 cases stained with calponin, all with caldesmon and 4 with smooth muscle actin and 6 with S100. ER, PR and c-erbB2 were negative in all cases. Anti-P53 antibody stained greater than 10% of cells in 2 cases. In contrast, of the 7 'control' cases, 5 were negative for all other myoepithelial markers. 2 cases showed focal or diffuse S100 positivity only, all other markers were negative. 2 were positive for c-erbB2 and 3 for P53 protein. 5 cases were both ER- and PR-positive.

CGH was carried out on these cases both on CK14-positive and, where applicable (4 cases), negative areas of the same tumour (Table 3) as well as in the 7 totally keratin 14-negative matched cases (Table 4). The CK14-positive areas of tumour, whether focal or diffuse, showed relatively few alterations, with a mean DNA copy number change of 3.0. Where there were focal CK14-negative areas within the tumour, these samples showed some overlap in genetic alterations with the CK14-positive areas of the tumour, but with further alterations (mean – 6.0). The 7 age- and grade-matched CK14-negative invasive ductal carcinomas-NST showed a molecular cytogenetic profile by CGH consistent with the extensive literature on such cases (mean – 8.3, Table 4). The difference in mean DNA copy number changes between

**Table 1** Antibodies used for immunohistochemistry

| Antibody | Manufacturer | Source | Dilution | Pre-treatment |
|---|---|---|---|---|
| CK14 | A. Menarini | mouse | 1:60 | 2 min pressure cooker |
| CK19 | Dako | mouse | 1:50 | microwave (Dako Target Retrieval Solution) |
| calponin | A. Menarini | mouse | 1:3000 | 2 min pressure cooker |
| caldesmone | A. Menarini | mouse | 1:100 | 2 min pressure cooker |
| SMA | Dako | mouse | 1:150 | no pre-treatment |
| S100 | Dako | rabbit | 1:2000 | 5 min chymotrypsin (0.1%, pH 7.8) |
| ER | Dako | mouse | 1:60 | 3 min pressure cooker |
| PR | Dako | mouse | 1:100 | 3 min pressure cooker |
| p53 | Dako | mouse | 1:40 | 2 min pressure cooker |
| c-erbB2 | Cambridge Bioscience | mouse | neat | 3 min pressure cooker |

© 2001 Cancer Research Campaign

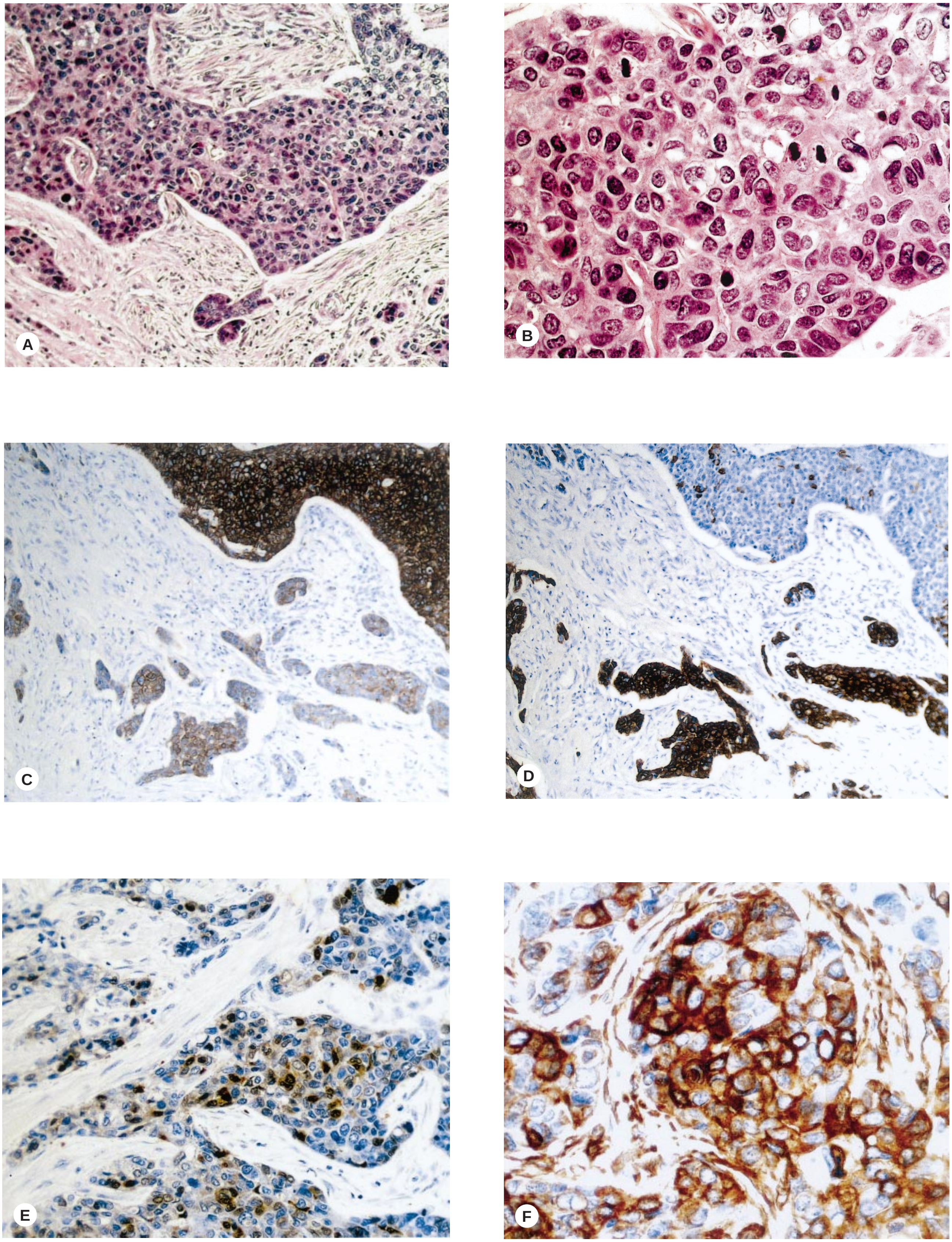

**Figure 1** Invasive ductal carcinoma with basaloid/myoepithelial cell differentiation, case 5. (**A**) Haematoxylin and eosin stained, × 100; (**B**) Haematoxylin and eosin stained, × 400 (**C**) Immunohistochemically stained for cytokeratin 14, × 100; (**D**) Immunohistochemically stained for cytokeratin 19, × 100; (**E**) Immunohistochemically stained for s100, × 200; (**F**) Immunohistochemically stained for SMA, × 200

 © 2001 Cancer Research Campaign

**Table 2** Immunohistochemical findings

| Case | CK14 | CK19 | Calponin | Caldesmone | SMA | S100 | ER | PR | ERBB2 | p53 |
|---|---|---|---|---|---|---|---|---|---|---|
| 1* | strong<br>10% | strong<br>100% | –ve | moderate<br>50% | –ve | strong<br>25% | –ve | –ve | –ve | –ve |
| 2 | strong<br>50–70% | strong<br>90–100% | strong<br>25–50% | weak<br>75% | strong<br>75% | –ve | –ve | –ve | –ve | –ve |
| 3 | strong<br>90–100% | –ve | –ve | strong<br>50–75% | –ve | strong<br>75–90% | –ve | –ve | –ve | –ve |
| 4 | strong<br>10% | –ve | –ve | strong<br>75% | –ve | strong<br>90–100% | –ve | –ve | –ve | positive |
| 5 | strong<br>10% | strong<br>50% | weak<br><10% | weak<br><10% | strong<br><10% | strong<br><10% | –ve | –ve | –ve | –ve |
| 6 | strong<br>90–100% | strong<br>90–100% | strong<br>25–50% | weak<br>50% | strong<br><10% | moderate<br>75% | –ve | –ve | –ve | positive |
| 7 | strong<br>10–20% | strong<br>50% | strong<br><10% | strong (c)<br>>75% | strong<br><10% | strong<br>>90% | –ve | –ve | –ve | –ve |
| 8 | –ve | strong<br>90–100% | –ve | –ve | –ve | weak<br>75% | positive | positive | –ve | –ve |
| 9 | –ve | strong<br>90–100% | not done | not done | not done | not done | positive | positive | positive | –ve |
| 10 | –ve | strong<br>90–100% | –ve | –ve | –ve | –ve | –ve | –ve | positive | positive |
| 11 | –ve | strong<br>90–100% | –ve | –ve | –ve | –ve | positive | positive | –ve | positive |
| 12 | –ve | strong<br>90–100% | –ve | –ve | –ve | –ve | positive | positive | –ve | –ve |
| 13 | –ve | strong<br>90–100% | –ve | –ve | –ve | strong<br>90–100% | –ve | –ve | –ve | positive |
| 14 | –ve | strong<br>90–100% | –ve | –ve | –ve | –ve | positive | positive | –ve | –ve |

*myosin – weak, 10–20%.

**Table 3** Clinicopathological and molecular cytogenetic data for the CK14-positive breast tumours. CGH results in italics denote overlaps between CK14 positive and negative areas of the same tumour

| Case | Age | LN | Microscopy | Size (cm) | Grade | CK14 IHC | CGH |
|---|---|---|---|---|---|---|---|
| 1 | 68 | N/A | invasive ductal carcinoma | 1.6 | 3 | focally positive<br>focally negative | *–17q24-qter*, *–19*, *–20p*, –Xp22<br>–16p, *–17q24-qter*, *–19*, *–20p*, –22q |
| 2 | 44 | 0/1 | invasive ductal carcinoma | 1.5 | 3 | diffusely positive | +3p24–p25, –16q24, –Xp21–p22 |
| 3 | 35 | 1/9 | invasive ductal carcinoma | 2.5 | 3 | diffusely positive | +8p21–p22 +20q12–q13 |
| 4 | 87 | N/A | invasive ductal carcinoma | 5 | 3 | focally positive<br>focally negative | *–16q*, *–17q24-qter*, –19p<br>+9q 33–q34, *–16q*, *–17q24-qter*, –19q, +20, +21q |
| 5 | 85 | N/A | invasive ductal carcinoma | 1.7 | 3 | focally positive<br>focally negative | –11p15, *–19q13*, –Xp22<br>+8q23–q24, –17q24-qter, *–19q13*, –20p13, +20q13, –22q, |
| 6 | 44 | 22/25 | invasive ductal carcinoma | 10 | 3 | diffusely positive | –16p13, –16q, –19 |
| 7 | 46 | N/A | invasive ductal carcinoma | 3.5 | 3 | focally positive<br>focally negative | *–16p*, *–17p13*, *–19q*<br>+7p21–p22–7p36, -8p24-pter, +8q24-qter, *–16p*, *–17p13*, *–19q* |

CK14-positive and CK14-negative tumours was significant using the one-sided *t*-test (95% confidence level).

### DISCUSSION

Invasive ductal carcinoma-NST, as determined morphologically, are thought to arise exclusively from the luminal epithelial cells in the breast A proportion of these tumours have been demonstrated to show a basal/myoepithelial cell phenotype by immunohistochemistry (Gusterson et al, 1982; Nagle et al, 1986; Dairkee et al, 1988; Guelstein et al, 1988; Gould et al, 1990; Wetzels et al, 1991; Malzahn et al, 1998; Tsuda et al, 1999) and patterns of gene expression (Perou et al, 2000). Here we have reported 7 cases of a grade III invasive carcinoma (6.6% of the unselected series of invasive ductal carcinomas) that immunophenotypically were characterized by positivity with keratin 14 as well as smooth muscle actin, calponin and caldesmone, all of which are markers of myoepithelial cells (Foschini et al, 2000). We have adopted the term of basaloid/myoepithelial cell phenotype for these tumours by analogy with prostate basal cells that are keratin 14 positive.

© 2001 Cancer Research Campaign 

**Table 4** Clinicopathological and molecular cytogenetic data for the CK14-negative breast tumours

| Case | Age | LN | Microscopy | Size (cm) | Grade | CK14 IHC | CGH |
|---|---|---|---|---|---|---|---|
| 8 | 56 | 0/15 | invasive ductal carcinoma | 2.5 | 3 | diffusely negative | –1p32-pter, –2p23-pter, +3p24, –3q27-qter, +6p21, –16p13, –17q24–q25 |
| 9 | 43 | N/A | invasive ductal carcinoma | 1.2 | 3 | diffusely negative | –1p32-pter, +1p22–p31, +6q13, –8p21, +8q21-q23, +9p13–p23, –9q33-qter, –12q23–q24, –16p, –17p13, –19 |
| 10 | 36 | 0/12 | invasive ductal carcinoma | 2.3 | 3 | diffusely negative | –1p36-pter, –16q22–q24, –17q25, –19p13, –21q13, –Xp22 |
| 11 | 87 | 3/8 | invasive ductal carcinoma | 3.5 | 3 | diffusely negative | +1q, –6p25, +6q14–q22, +8q, –16q23–q24, –17p13, +17q23–q24, +18, –19p13, +22q |
| 12 | 83 | N/A | invasive ductal carcinoma | 1.0 | 3 | diffusely negative | +1p13, +1q43–q44, –6p25, +6q14–q22 +8, –14q22–q32, –16p13, +16q24, +17p13, –17q24, +20q13 |
| 13 | 44 | N/A | invasive ductal carcinoma | 0.5 | 3 | diffusely negative | –15q26, –17p13, –19p13 |
| 14 | 46 | 13/20 | invasive ductal carcinoma | 1.5 | 3 | diffusely negative | –6q24-qter, +7q11, +10p13–p15, +16p13, –17p13, +17q22–q24, +18p11, –18q22–q23, –19q, +20q13, +21q |

Myoepithelial cells both in salivary and breast glands also contain this specific type of keratin in addition to smooth muscle actin, calponin and caldesmon. A similar type of lesion had been reported as poorly differentiated myoepithelial rich carcinomas (Damiani et al, 1997).

Previous work in our laboratory has demonstrated a specific pattern of genetic alterations associated with conventional myoepithelial cell carcinomas, with common losses at 11q, 16p, 16q, 17p, and 17p part of only a small total number of DNA copy number changes (Jones et al, 2000). The present invasive ductal carcinomas showing a basal/myoepithelial phenotype showed a pattern and number of alterations which was similar to that of pure myoepithelial carcinomas, but did not show changes commonly associated with classical ductal carcinomas (losses at 8p and gains at 8q, 17q, 20q). Some of these tumours also showed areas of focal CK14-negativity. Such regions showed alterations found in myoepithelial carcinomas as well as further alterations, consistent with CGH data from the literature on invasive ductal breast carcinoma (Nishizaki et al, 1997; Schwendel et al, 1998; Tirkkonen et al, 1998; Buerger et al, 1999; Roylance et al, 1999) as well as the present control-matched cases. The overlap in DNA copy number changes between the CK14-positive and -negative areas from the same case suggests a possible origin from a common precursor cell with subsequent divergent differentiation.

All of the tumours exhibiting a myoepithelial phenotype were histological grade III, which in is keeping with literature reports of an association with high grade of malignancy. A recent paper (Tsuda et al, 2000) highlighted the aggressive nature of tumours with a myoepithelial phenotype, demonstrating that invasive ductal carcinomas with a basal phenotype and large, central acellular zones were an indicator of high risk of brain and lung metastases and of death by cancer independent of nodal status and tumour size.

The findings that ‘ordinary’ invasive ductal carcinomas-NST with a basal phenotype behave in a clinically aggressive fashion (Tsuda et al, 2000) as well as having a specific pattern of genetic alterations similar to pure myoepithelial carcinomas highlights the fact that grade III ductal carcinomas contain a subset of tumours with different biological characteristics and hence prognosis for the patient. Although we have studied a small number of samples to date, the lack of ER and PR expression and p53 positivity in some cases is consistent with this view. It is interesting that all these high-grade tumours were c-erbB2-negative. This combination of phenotype (ER, PR, c-erbB2-negative) is similar to that seen in BRCA1-associated tumours (Johannsson et al, 1997). The number of cases studied is too small to make generalizations but clearly warrants further investigation of these interesting subset of grade III ductal carcinomas These data also indicate that the spectrum of myoepithelial tumours in the breast is larger than previously appreciated and includes the subset described here. This will have to be added to the ‘classical’ range of myoepithelial cell carcinomas (Foschini and Eusebi, 1998).

### ACKNOWLEDGEMENTS

Supported in part by the Ludwig Institute for Cancer Research, the Sydney and Phyllis Goldberg Memorial Charitable Trust. The image capture equipment was funded by The Wellcome Trust (grant ref: 039938). We are grateful to Damian Counsell for statistical analysis. We would like to thank Dr K McCarthy and Mr Modi for allowing us to use their case in this study. We are grateful to Professor A Munro Neville, Professor Niki Agnantis and Dr Mike O’Hare for their encouragement and support.

### REFERENCES

Accurso A, Donofrio V, Insabato L and Mosella G (1990) Adenomyoepithelioma of the breast. A case report. *Tumori* **76**: 606 – 610

Barnes DM, Harris WH, Smith P, Millis RR and Rubens RD (1996) Immunohistochemical determination of oestrogen receptors: comparison of different methods of assessment of staining and correlation with clinical outcome of breast cancer patients. *Br J Cancer* **74**: 1445–1451

Buerger H, Otterbach F, Simon R, Schäfer K-L, Poremba C, Diallo R, Brinkschmidt C, Dockhorn-Dworniczak B and Boecker W (1999) Different genetic pathways in the evolution of invasive breast cancer are associated with distinct morphological subtypes. *J Pathol* **189**: 521–526

Dairkee SH, Puett L and Kackett AJ (1988) Expression of basal and luminal epithelium-specific keratins in normal, benign and malignant breast tissue. *J Natl Cancer Inst* **80**: 691–695

Damiani S, Riccioni L, Pasquinelli G and Eusebi V (1997) Poorly differentiated myoepithelial cell rich carcinoma of the breast. *Histopathology* **30**: 542–548

Desautels JE (1990) Malignant myoepithelioma of the breast: a case report. *Can Assoc Radiol J* **41**: 387–388

 © 2001 Cancer Research Campaign

Ellis IO, Dowsett M, Bartlett J, Walker R, Cooke T, Gullick W, Gusterson B, Mallon E and Barrett Lee P (2000) Recommendations for HER2 testing in the UK. *J Clin Pathol* **53**: 890–892

Erlandson RA and Rosen PP (1982) Infiltrating myoepithelioma of the breast. *Am J Surg Pathol* **6**: 785–793

Eusebi V, Casadei GP, Bussolati G and Azzopardi JG (1987). Adenomyoepithelioma of the breast with a distinctive type of apocrine adenosis. *Histopathology* **11**: 305–315

Foschini MP and Eusebi V (1998) Carcinomas of the breast showing myoepithelial cell differentiation. *Virchows Arch* **432**: 303–310

Foschini MP, Scarpellini F, Gown AM, Eusebi V. Differential expression of myoepithelial markers in salivary, sweat and mammary glands. *Int J Surg Pathol 2000*; **8**: 29–37

Gould VE, Koukoulis GK, Jansson DS, Nagle RB, Franke WW and Moll R (1990) Coexpression patterns of vimentin and glial filament protein with cytokeratins in the normal, hyperplastic and neoplastic breast. *Am J Pathol* **137**: 1143–1155

Guelstein VI, Tchypysheva TA, Ermilova VD, Litvinova LV, Troyanovsky SM and Bannikov GA (1988) Monoclonal antibody mapping of keratins 8 and 17 and of vimentin in normal human mammary gland, benign tumors, dysplasias and breast cancer. *Int J Cancer* **42**: 147–153

Gusterson BA, Warburton MJ, Mitchell D, Ellison M, Neville AM and Rudland PS (1982) Distribution of myoepithelial cells and basement membrane proteins in the normal breast and in benign and malignant breast diseases. *Cancer Res* **42**: 4763–4770

Johannsson OT, Idvall I, Anderson C, Borg A, Barkardottir RB, Egilsson V and Olsson H (1997) Tumour biological features of BRCA1-induced breast and ovarian cancer. *Eur J Cancer* **33**: 362–371

Jones C, Foschini MP, Chaggar R, Lu Y-J, Wells D, Shipley JM, Eusebi V and Lakhani SR (2000) Comparative genomic hybridization analysis of myoepithelial carcinoma of the breast. *Laboratory Investigation* **80**: 831–836

Lakhani SR, O'Hare MJ, Monaghan P, Winehouse J, Gazet JC and Sloane JP (1995) Malignant myoepithelioma (myoepithelial carcinoma) of the breast: a detailed cytokeratin study. *J Clin Pathol* **48**: 164–167

Lakhani SR, Chaggar R, Davies S, Jones C, Collins N, Odel C, Stratton MR and O'Hare MJ (1999) Genetic alterations in 'normal' luminal and myoepithelial cells of the breast. *J. Pathology* **189**: 496–503

Malzahn K, Mitze M, Thoenes M and Moll R (1998) Biological and prognostic significance of stratified epithelial cytokeratins in infiltrating ductal breast carcinomas. *Virchows Arch* **433**: 119–129

Nagle RB, Bocker W, Davis JR, Heid HW, Kaufmann M, Lucas DO and Jarasch ED (1986) Characterization of breast carcinomas by two monoclonal antibodies distinguishing myoepithelial from luminal epithelial cells. *J Histochem Cytochem* **34**: 869–881

National Coordinating Group for Breast Screening Pathology (1995) Pathology reporting in breast cancer screening. NHS Breast Screening Programme Publication No.3, NHSBSP Publications, Sheffield

Nishizaki T, DeVries S, Chew K, Goodson III WH, Ljung B-M, Thor A and Waldman FM (1997) Genetic alterations in primary breast cancers and their metastases: direct comparison using modified comparative genomic hybridization. *Genes Chromosomes Cancer* **19**: 267–272

Perou CM, Sørlie T, Eisen MB, van de Rijn M, Jeffrey SS, Rees CA, Pollack JR, Ross DT, Johnsen H, Akslen LA, Fluge Ø, Pergamenschikov A, Williams C, Zhu SX, Lønning PE, Børresen-Dale A-L, Brown PO, Botstein D (2000) Molecular portraits of human breast tumours. *Nature* **406**: 747–752

Roylance R, Gorman P, Harris W, Liebmann R, Barnes D, Hanby A and Sheer D (1999) Comparative genomic hybridization of breast tumors stratified by histological grade reveals new insights into the biological progression of breast cancer. *Cancer Res* **59**: 1433–1436

Schwendel A, Richard F, Langreck H, Kaufmann O, Lage H, Winzer KJ, Petersen I and Dietel M (1998) Chromosome alterations in breast carcinomas: frequent involvement of DNA losses including chromosomes 4q and 21q. *Br. J. Cancer* **78**: 806–811

Shiraishi T, Nakayama T, Fukutome K, Watanabe M and Murata T (1999) Malignant myoepithelioma of the breast metastasising to the jaw. *Virchows Arch* **435**: 520–523

Tavassoli FA (1991) "Myoepithelial lesions of the breast. Myoepitheliosis, adenomyoepithclioma, and myoepithelial carcinoma. *Am J Surg Pathol* **15**: 554–568

Thorner PS, Kahn HJ, Baumal R, Lee K and Moffatt W (1986) Malignant myoepithelioma of the breast. An immunohistochemical study by light and electron microscopy. *Cancer* **57(4)**: 745–750

Tirkkonen M, Tanner M, Karhu R, Kallioniemi A, Isola J and Kallioniemi OP (1998) Molecular cytogenetics of primary breast cancer by CGH. *Genes Chromosomes Cancer* **21**: 177–184

Tsuda H, Takarabe T, Hasegawa T, Murata T and Hirohashi S (1999) Myoepithelial differentiation in high-grade invasive ductal carcinoma with large central acellular zones. *Hum Pathol* **30**: 1134–1139

Tsuda H, Takarabe T, Hasegawa F, Fukotomi T and Hirohashi S (2000) Large, acellular zones indicating myocpithelial tumor differentiation in high-grade invasive ductal carcinomas as markers of predisposition to lung and brain metastases. *Am J Surg Pathol* **24**: 197–202

Wells D, Sherlock JK, Handyside AH and Delhanty JDA (1999) Detailed chromosomal and molecular genetic analysis of single cells by whole genome amplification and comparative genomic hybridization. *Nucleic Acids Res* **27**: 1214–1218

Wetzels RHW, Kuijpers HJH, Lane EB, Leigh IM, Troyanovsky SM, Holland R, van Haelst UJGM and Ramaekers FCS (1991) Basal cell-specific and hyperproliferation-related keratins in human breast cancer. *Am J Pathol* **138**: 751–763

© 2001 Cancer Research Campaign 
